# Health-related quality of life and pain in children and adolescents: a school survey

**DOI:** 10.1186/s12887-017-0927-4

**Published:** 2017-07-24

**Authors:** Kristin Haraldstad, Knut-Andreas Christophersen, Sølvi Helseth

**Affiliations:** 1 Faculty of Health- and Sport Sciences, University of Agder, P.O box 422, 4604 Kristiansand, Norway; 2 Faculty of Health, Oslo and Akershus University College of Applied Sciences, P.O box 4 St Olavs Plass, 0130 Oslo, Norway; 3 Faculty of Social Sciences, University of Oslo, P.O box 1084 Blindern, 0317 Oslo, Norway

**Keywords:** Adolescents, Children, HRQoL, Pain, Public health, School survey

## Abstract

**Background:**

Pain problems are common in children and adolescents. Measures of health-related quality of life (HRQoL) can be used to assess children’s subjective perspectives of pain experience and its impact on their life. The aims of the study were to describe HRQoL and the prevalence of pain in a nonclinical population of children and adolescents, and to analyze the relationships between HRQoL, pain, sex, and age in a sample of children and adolescents aged 8–18 years.

**Methods:**

This cross-sectional study involved a cluster sample of 20 randomly selected schools drawn within a region of Norway. The final study sample included 1099 children and adolescents. We measured HRQoL using the generic questionnaire KIDSCREEN-52 and pain using questions from the Lübeck Pain-Screening Questionnaire. Multiple regression was used to analyze relationships between HRQoL and sex, age, and pain.

**Results:**

The response rate was 74%. A large percentage of the sample, 60%, reported pain, and girls reported significantly more pain than boys, 76% of the girls in the age group 16–18 years reported pain**.** The KIDSCREEN-52 scores differed between girls and boys, and on average, girls reported a significantly lower HRQoL than boys on most dimensions. Pain problems were associated with lower HRQoL, and older girls were most impaired by pain.

**Conclusions:**

The findings from this study indicate that pain problems are highly prevalent in children, and more prevalent in girls than in boys. HRQoL was impaired for all 10 dimensions of the KIDSCREEN-52 in children with pain. The subscales self-perception, psychological well-being, mood, relationship with parents, and school environment were most affected.

## Background

Pain problems among children and adolescents are a serious health concern, and studies show that 15–35% of children in a nonclinical population experience a persistent or chronic pain condition [1–3]. The prevalence of pain increases with age, and older children report more pain than do younger children. Headache is the most commonly reported pain, followed by neck and shoulder pain, abdominal pain, backache, and limb pain, and several children report pain in multiple places [4–6]. Traditionally, more girls report pain than boys [2, 6, 7]. Pain can influence daily life in different ways and is linked to poor school performance, problems in social activities, sleep problems, and reduced quality of life [1, 8]. Moreover, pain problems may contribute to school absenteeism, school impairment, and problems meeting school demands [9, 10].

The high prevalence is a cause for concern given that pain problems early in life may contribute to pain problems later in life [11–13]. Pain problems in childhood should also be viewed in relation to research showing that backache, limb pain, and other musculoskeletal complaints are frequent causes of sickness certification and disability pensions in adult populations, and are becoming increasingly so among young adults [14, 15]. These findings indicate that pain can also have serious long-term effects [11, 16].

Pain is a subjective and individual experience and is often defined as “whatever the experiencing person says it is, existing whenever he says it does” [17]. This definition stresses that pain is a subjective concept and the importance of accepting the person’s perception and experience of pain.

Pain and physical complaints of children and adolescents may be symptoms of underlying causes and may be related to several psychological, social, and physical factors. The complexity of pain etiology calls for a biopsychosocial understanding and approach [18]. Health-related quality of life (HRQoL) is a construct that is used to assess children’s subjective perspectives of health and is often used to examine how health problems such as pain can interfere with daily life [19–21]. HRQoL is generally conceptualized as a multidimensional construct that includes the individual’s subjective perspectives on the physical, psychological, social, and functional aspects of health [22]. The multidimensionality of HRQoL measures may provide researchers and clinicians with information about the impact of a health condition, e.g. pain, on different aspects of quality of life, and may serve as a framework for identifying and develop strategies to promote quality of life, and develop tailored interventions [20, 22–24].

Understanding of pain in children has increased in recent years, and it is recognized that children with pain can experience various problems related to their pain. However, relatively little is known about the associations between pain and HRQoL. There are studies of pain and HRQoL in children [9, 19–25], however, some of the studies have used one global score to measure HRQoL, which make it impossible to identify which dimensions of HRQoL are most affected by pain, and, and some studies focusing on children with chronic pain recruited from pain clinics [9, 19–21, 25]. Clearly, more knowledge is needed on pain and HRQoL in a non-clinical population of children and adolescents.

The aims of this study were to first describe HRQoL and the prevalence of pain in children and adolescents and, second, to analyze the relationships between HRQoL (dependent variable) and pain, sex, and age (independent variables) in a representative sample of children and adolescents aged 8–18 years.

## Methods

### Sample and data collection

This cross-sectional study was performed in a region in the eastern part of Norway with around 1.7 million inhabitants (36% of the total Norwegian population) and a child/adolescent population (8–18 years) of around 230,000. Statistics Norway was responsible for planning and conducting the sampling. First, all schools in the region were stratified into 10 strata according to the population density, school size, and school level. Two schools were chosen randomly from each stratum. Seven of the invited schools declined to participate and were replaced by schools selected according to the same criteria. From the sample of 20 schools, classes that covered grades 3, 5, 7, and 9 in elementary school and grades 1 and 3 in secondary school were selected.

The children were invited to participate after their school had agreed to participate. A total of 1675 children and adolescents aged 8–18 years were recruited. The exclusion criterion was an inability to read and write Norwegian. The data collection took place over a 6-month period from October to April. The children and adolescents, and their teachers were given verbal and written information at the school by the investigator 1 week before the study took place. The students received standard information about the study and a written consent form for their parents to read and sign. The study was reviewed and approved by the Regional Research Ethics Committee of Norway (REK. Sør S-06143).

The self-report instruments were administrated and completed in the classrooms during school time. The investigator and teacher were present and were allowed to assist the children if they needed. For grade 3 in elementary school, the questionnaire was read aloud to the whole class by the investigator. Those children who were absent from school on the day of the study were not included in the study.

### Instruments

#### Demographic variables

The first part of the questionnaire recorded demographic details such as nationality, sex, and date of birth.

#### Health-related quality of life

To measure *HRQoL*, the KIDSCREEN-52 questionnaire was administered. The KIDSCREEN-52 questionnaire is a generic HRQoL instrument that is based on a multidimensional HRQoL construct [26]. It is intended to assess HRQoL from the child’s or adolescent’s perspective and focuses on the physical, mental, and social dimensions of well-being. The KIDSCREEN instrument aims to identify children and adolescents at risk for problems related to health. The instrument includes 52 items, which are rated on a five-point Likert scale. The scale indicates either the frequency of certain behaviors or feelings (1 = never, 2 = seldom, 3 = sometimes, 4 = often, and 5 = always) or the intensity of an attitude (1 = not at all, 2 = slightly, 3 = moderately, 4 = very, and 5 = extremely). The timeframe refers to the previous week. The 52 items are distributed into the following 10 aspects or dimensions: physical well-being (five items), psychological well-being (six items), moods and emotions (seven items), self-perception (five items), autonomy (five items), relationship with parents and home life (six items), social support and peers (six items), school environment (six items), social acceptance/bullying (three items), and financial resources (three items).

The scale for negatively worded items was reversed, and missing values were substituted using the mean of the nonmissing items; however, no score was computed if more than one item per scale was left unanswered [22, 27]. The three-item scales required that all items were completed. The dimension score was then transformed linearly to a 0–100-point scale, with 100 indicating the best HRQoL and 0 the worst [22]. The instrument has been shown to have adequate reliability and validity in several countries, including Norway [27, 28].

#### Pain

The *pain* instrument was based on the Lübeck Pain-Screening Questionnaire [29]. This is a structured self-report questionnaire that evaluates the prevalence and consequences of pain during the previous 3 months. The questionnaire has been translated and used in Norway [1]. Previous studies have demonstrated that the feasibility, content, and face validity of the Lübeck Pain-Screening Questionnaire are satisfactory [1, 29]. In our study, we used three pain-related questions from the questionnaire to construct a pain variable. The pain variable was created as the average of the three indicators frequency, duration, and intensity (“How long have you had pain?”, “How often have you had this pain?”, and “How strong/intense is the pain?”). Each indicator was measured on a scale of 0–6, where 0 indicates that the student experiences little pain and 6 that the student experiences a lot of pain. For children who answered these questions, the mean value for all indicators was recorded. Children who did not answer these questions but answered as having no pain at all on the fourth question received a pain score of zero. Cronbach’s alpha for the pain indicators was 0.92.

### Data analysis

The analyses were conducted using SPSS (version 22.0). Descriptive statistics are presented as frequencies and percentages for the variables age, sex, and pain. To describe HRQoL and pain in different age groups, age was grouped into three categories in Table 3.

Pearson’s product moment correlation was used to examine the bivariate relationships between each of the 10 dimensions of KIDSCREEN-52 scales and the independent variables sex, age and pain respectively. Nine of the ten dimensions had skewness between ±1.5, and eight had kurtosis between ±1. This indicate that these variables are approximately normally distributed. However, the bullying dimension (no 10) had skewness −2.43 and kurtosis 7.41, so the standard error for this dimension may be somewhat impaired. To examine further how pain, age, and gender were associated with HRQoL, multiple linear regression analyses were performed with each KIDSCREEN subscale as dependent variables. In these analyses age and pain variables were considered continuous. Multilevel analysis was considered because data were collected at schools. However, we decided to use linear regression for three reasons. First, the grouping was weak for eight scales (.01 < ICC < .10) and only moderate for two of the scales (ICC ≥ .16). Second, the level 1 variables explained only a small amount of level 1 (1% to 9%), but a large part (52% to 91%) of the level 2 variance. This is because school class and age are highly collinear and therefore a bit misleading. Third, our focus was on explained variance of HRQoL, and not on regression coefficients. Internal consistency reliability for multi-item scales was estimated using Cronbach’s alpha with a value ≥0.70 considered satisfactory for group comparisons [30].

## Results

Of the 1675 invited children and adolescents, 258 (15%) did not return the written permission from their parents, 148 (9%) were absent from school on the day of the study, and 31 (2%) did not want to participate. Hence, the final sample comprised 1238 children and adolescents, with an overall response rate of 74%. After excluding children with missing values on the KIDSCREEN scales, the data for 1099 children and adolescents were eligible for analysis.

Tables 1 and 2 presents the characteristics of the sample and descriptive statistics for the ten dimensions of KIDSCREEN-52, sex, age and pain. The study population comprised 1099 children and adolescents: 45.9% boys and 54.1% girls. The largest group of study participants was aged between 12 and 15 years (44.2%), and mean age was 13 years.

**Table 1:** Characteristics of the sample, by age, sex and nationality, *n* = 1099

	N (%)
Age	
8–11 yrs	315 (28.7)
12–15 yrs	486 (44.2)
16–18 yrs	298 (27.1)
Sex	
Girls	595 (54.1)
Boys	504 (45.9)

**Table 2:** Descriptive statistics for kidscreen, sex, age and pain, *n* = 1099

	Min	Max	Mean	SD	Skewness	Kurtosis
Physical well-being	0	100	66.73	20.32	−0.38	−0.47
Psychological well-being	8	100	77.77	17.73	−1.05	0.99
Mood	0	100	81.60	15.00	−1.47	2.70
Self-perception	0	100	74.34	20.76	−0.85	0.09
Autonomy	0	100	70.45	20.48	−0.61	−0.07
Parents relation	0	100	77.81	18.79	−0.98	0.68
Financial resources	0	100	76.11	23.93	−0.95	0.24
Social support a peers	0	100	74.06	17.78	−0.69	0.26
School environment	0	100	66.80	20.09	−0.36	−0.25
Bullying	0	100	90.20	15.65	−2.43	7.41
Sex	0	1	0.46	0.50	0.17	−1.98
Age	7	19	13.16	2.87	0.26	−0.84
Pain	0.00	6.00	2.04	1.92	0.23	−1.43

### HRQoL and the prevalence of pain

Table 3 presents the HRQoL and pain prevalence in all 10 domains of the KIDSCREEN-52 according to sex and age. Age was grouped into three categories: 8–11, 12–15, and 16–18 years. Sixty percent of the children reported pain within the previous 3 months, and significantly more girls (64.2%) than boys (53.8%) reported pain (*p <* 0.01).

**Table 3:** Health related quality of life measured with KIDSCREEN-52 and pain by sex and age, *n* = 1099

		Girls					Boys					**Total**		**Total**
KIDSCREEN-scales	No of items	8-11 yrs. *n* = (170)	12-15 yrs. *n* = (262)	16-18 yrs. *n* = (163)	Total *n* = 595	p^a^	8-11 yrs. *n* = (145)	12-15 yrs. *n* = (224)	16-18 yrs. *n* = (135)	Total *n* = 504	p^a^	p^b^	p^c^	*n* = 1099
Physical well-being	5	69.94	66.43	54.26	64.10	< 0.001	72.62	71.05	64.85	69.84	0.002	< 0.001	< 0.001	66.73
Psychological well-being	6	82.89	76.70	69.66	76.54	< 0.001	82.93	79.87	74.14	79.22	< 0.001	.013	< 0.001	77.77
Mood	7	82.46	82.28	73.99	80.06	< 0.001	83.89	83.45	82.86	83.42	.814	< 0.001	< 0.001	81.60
Self-perception	5	82.53	67.61	56.96	68.96	< 0.001	86.69	80.76	74.19	80.70	< 0.001	< 0.001	< 0.001	74.34
Autonomy	5	74.56	72.42	55.46	68.39	< 0.001	75.41	74.42	67.59	72.88	< 0.001	< 0.001	< 0.001	70.45
Parents relation	6	82.25	77.24	70.40	76.80	< 0.001	83.22	78.46	75.40	79.01	< 0.001	.052	< 0.001	77.81
Financial resources	3	71.86	76.27	77.81	75.43	.059	71.03	79.35	79.20	76.92	.002	.306	< 0.001	76.11
Social support and peers	6	77.89	75.91	72.83	75.63	.030	75.55	71.63	69.57	72.21	.015	.001	0.001	74.06
School environment	6	79.31	67.10	57.39	67.93	< 0.001	75.37	65.63	54.60	65.48	< 0.001	.044	< 0.001	66.80
Bullying	3	86.76	90.17	95.60	90.69	< 0.001	85.34	90.07	93.46	89.62	< 0.001	.259	< 0.001	90.20
Pain n/%		72 / 42.4	186/71.0	124/76.1	382/64.2	< 0.001	65/44.8	119/53.1	87/64.4	271/53.8	0.004	< 0.001	< 0.001	653/59.6

p^a^ age differences within sex (f-test); p^b^ sex differences (t-test); p^c^ age differences (f-test)

The KIDSCREEN-52 scores differed between girls and boys. On average, girls reported a significantly lower HRQoL than boys on most dimensions (*p <* 0.01) except for relationship with parents, financial resources, and bullying. For girls, the lowest scores were reported for physical well-being, self-perception, school, and autonomy. For boys, the lowest scores were for the school environment and physical well-being. HRQoL decreased significantly with age for both boys and girls, except for the subscales of relationship with parents, financial resources, bullying, and the school environment.

### Relationship between HRQoL, age, sex, and pain

Table 4 shows the significant results (*p* < 0.05) for the total R^2^ values and the unique contributions of each of the independent variables. In model 1, R^2^ varied between 0.013 and 0.217, meaning that sex and age accounted for 1–22% of the variance in the KIDSCREEN-52 subscales. Age made the largest contribution in all subscales. However, sex predicted 8% of the variance in the self-perception subscale, which had the largest contribution to the effect of sex in this model. Adding the interaction between sex and age increased the predicted variance significantly in the subscales physical well-being, mood, self-perception, and autonomy. This indicates that the effect of age is different for boys and girls, girls are more impaired than boys.

**Table 4:** Unique and total predicted variance of HRQOL measured with KIDSCREEN-52 due to sex. Age. pain and interaction between sex and age, *n* = 1099

	Pearson’s r			Model 1			Model 2		Model 3	
Scale	Sex	Age	Pain	R^2^	Sex^a^	Age^a^	R^2^	Interaction^a^	R^2^	Pain^a^
Physical well-being	0.141	−0.239	−0.199	0.076	0.019	0.056	0.082	0.006	0.092	0.016
Psychological well-being	0.075	−0.259	−0.257	0.072	0.005	0.067	0.075		0.107	0.035
Mood	0.112	−0.152	−0.266	0.035	0.012	0.023	0.046	0.011	0.085	0.050
Self-perception	0.282	−0.375	−0.301	0.217	0.076	0.137	0.235	0.018	0.248	0.031
Autonomy	0.109	−0.273	−0.207	0.085	0.011	0.074	0.098	0.012	0.102	0.016
Parents relation		−0.220	−0.244	0.052		0.048	0.053		0.086	0.035
Financial resources		0.109	−0.090	0.013		0.012	0.013		0.028	0.015
Social support and peers	−0.096	−0.128	−0.109	0.026	0.010	0.017	0.026		0.034	0.008
School environment	.0.061	−0.409	−0.272	0.171	0.004	0.168	0.172		0.203	0.031
Bullying		0.193	−0.097	0.038		0.037	0.038		0.064	0.025

Only significant (*p* < .05) results shown in the table

^a^Unique contribution

Pearson’s r: Bivariate correlations between HRQoL and sex. Age and pain respectively

Model 1: Sex and age. With the unique contributions of sex and age in addition to total R^2^. correlation sex and age. *r* = .02 (*p* = .615)

Model 2: Sex. age and interaction with the unique contribution of the interaction in addition to total R^2^

Model 3: Sex. age and pain with the unique contribution of pain in addition to total R^2^

Adding pain resulted in small changes in the predicted variance, however pain contributed significantly to explain variations in the subscales. As shown in Table 4, pain correlated negatively and moderately with the HRQoL subscales. Impaired HRQoL was prevalent in children with pain for all 10 dimensions of the KIDSCREEN-52 questionnaire. The children and adolescents with pain had a substantially lower HRQoL than did their peers who reported no pain. The subscales self-perception, psychological well-being, mood, autonomy and school environment were most affected.

## Discussion

The current study describes HRQoL and the prevalence of pain, and analyzes the associations between HRQoL, pain, sex, and age in a nonclinical population of children and adolescents. The results confirm the findings of earlier studies and show that pain is a common problem in young people. More than 60% of the children in this study reported pain in the previous 3 months. More girls than boys reported pain, and the prevalence of pain was highest in girls in the age group 16–18 years.

These results are consistent with those reported by other studies [4, 5, 7, 9, 20, 31, 32]. A Danish study of adolescents aged 12–19 years found a pain prevalence of 61%. The Norwegian HUNT study reported a pain prevalence of 44%, (pain once a week the last three months) among adolescents in a nonclinical population aged 13–18 years [7]. A systematic review of 32 studies on chronic pain in children found a high prevalence of chronic and recurrent pain in nonclinical populations and an increasing prevalence with age. The finding from this review indicated that most types of pain are more prevalent in girls than in boys. In the study, prevalence was described in relation to type of pain, and it is therefore difficult to make general conclusions regarding prevalence, however, the results indicated that pain is overwhelmingly prevalent in children, and should be regarded as a major health concern [4].

The prevalence varies between studies because of the use of different methods, age groups, and use of questionnaires, which makes it difficult to compare the results. However, most studies have shown a trend toward a high prevalence of pain in children and adolescents, especially in adolescent girls, and that pain is an increasing problem [4, 31–33].

The high prevalence of pain is worrying, and one of the aims of our study wasto examine the association with HRQoL and how children and adolescents with pain perceive their quality of life.

The results from the HRQoL analyses are consistent with previous findings showing that HRQoL varies between sexes on most subscales; that is, girls have poorer HRQoL than boys. However, the greatest variation in HRQoL is seen between different age groups, and age contributes significantly to explaining the variations in all of the HRQoL subscales. Overall, increasing age is associated with decreasing HRQoL. Earlier studies of HRQoL in children and adolescents have found sex and age differences [34, 35, 36]. Physical and mental well-being, in particular, seem to deteriorate with age and more so for girls than for boys. These changes may reflect the developmental challenges that adolescents experience, including physical, psychological, vocational, and social changes [36, 37].

The results from the bivariate analysis show that having pain was associated with a lower HRQoL in all subscales of the KIDSCREEN-52. Moreover, the interaction between sex and age increase the explained variance in the scales physical, mood, self-perception, and autonomy. This result is significant but rather small, however it indicates that the effect of age is different for boys and girls.

Even though pain was significantly associated with lower HRQoL in all KIDSCREEN-52 subscales, our analysis shows that adding pain into the model resulted in small changes in the explained variance; that is, pain was not found to be a strong explanatory factor for variations in HRQoL. This is in line with a study of American children (mean age 14 years) with chronic pain, which showed that pain did not account for the significant variance in HRQoL after controlling for demographics [19]. However, in our study, pain contributed significantly to explain the variations in the domains of psychological well-being, mood and emotions, self-perception, relationship with parents, and school environment. Our results indicate that children with pain are generally less happy, have a less positive perception of themselves, are less satisfied with their relationship with parents, and are less positive about going to school.

It is notable that children with pain had a low score on the school dimension in the KIDSCREEN-52, which includes satisfaction with one’s own ability to perform at school and general feelings about school. Similar results were reported in a Canadian study, in which children with pain were more likely to report fair or poor self-rated health, psychological health complaints, and greater unhappiness with school experiences [32, 33]. School and friends are significant arenas for social development, especially during adolescence. Children with pain may be more absent from school and miss theoretical and social experiences, which are fundamental to healthy development. A study of adolescents with chronic diseases found an increased risk of sickness and disabilities later in young adulthood [37]. In the current study, the self-perception and mood scores were low in children with pain and were lowest for older girls. Earlier studies indicate that girls are more affected by health problems and have more worries than do boys [38]. The score on the relationship with parents was low, and this subscale reflects the extent to which a child feels supported by his/her family [28]. Parents play an important role in helping and supporting how their children experience pain so that it does not lead to chronic pain. Pain is a subjective experience, and parents may differ in their sensitivity and ability to perceive and support their children’s complaints [39].

The results of our study are consistent with previous research demonstrating the importance of psychosocial factors to HRQoL among children with pain [21]. A few other studies have reported an association between pain and HRQoL in a nonclinical population. In a Swedish study of children aged 9 and 12 years, young schoolchildren with recurrent pain had a considerably impaired HRQoL. Pain was measured using questions from the international World Health Organization Health Behaviour in School-aged Children study. HRQoL impairment was twice as common in children with recurrent pain as in children without pain [24]. A Danish study among 4000 adolescents aged 12–19 years, found an association between pain problems and lower HRQoL; however, HRQoL was measured with the EQ. 5D questionnaire, and no single dimensions could be identified [25].

Our findings emphasize the vulnerability of adolescents, especially adolescent girls. Adolescence is a period when many changes occur over a relatively short time, and adolescents are faced with many physical, psychological, and social changes. Pain problems can negatively influence a young person’s life during this vulnerable period in life, and should be regarded as a significant health concern [9, 40, 41].

### Strengths and limitations

There are several strengths and limitations of this study. The relatively high response rate strengthens our ability to generalize from the results. The large sample size ensures that the results are representative of children in the eastern part of Norway. The Norwegian school system is fairly homogeneous, and the findings would probably be similar for this age group (8–18 years) in other Norwegian regions.

One limitation is that this was a cross-sectional study and, therefore, it is not possible to make substantial causal inferences. Measures of subjective symptoms, such as pain and the causes of pain, in children and adolescents may be influenced by other factors. Moreover, for younger children, it may be difficult to read and complete a questionnaire that requires them to remember information from the preceding 3 months (Lübeck Pain-Screening Questionnaire). A long recall period may weaken the content validity. However, the KIDSCREEN-52 has a recall period of 1 week, which is more appropriate [22, 28]. The different recall periods in the two questionnaires used may have been difficult, especially for the younger children. There was no additional sampling of children and adolescents who were absent on the day of the study. Children with health problems are more likely to be absent from school, and some of them might have been absent because of pain. However, the overall response rate of 74% is considered satisfactory [42].

## Conclusions

The findings from this study indicate that pain problems are highly prevalent in children and adolescents, and, and more prevalent in girls than in boys. Further, there is evidence that pain experienced by children and adolescents in a school sample affects all dimensions of HRQoL negatively. In KIDSCREEN 52, the subscales self-perception, psychological well-being, mood, relationship with parents, and school environment were most affected.

Early intervention and prevention of pain problems should be of great concern, especially for those involved in school health care. It is important to provide young people with strategies to manage their own health and to prevent negative consequences of pain problems. Further studies are required, especially longitudinal studies, to investigate the relationship between pain in childhood and later HRQoL.

## Abbreviations

HRQoL: Health-related quality of life
ICC: Intraclass correlation
